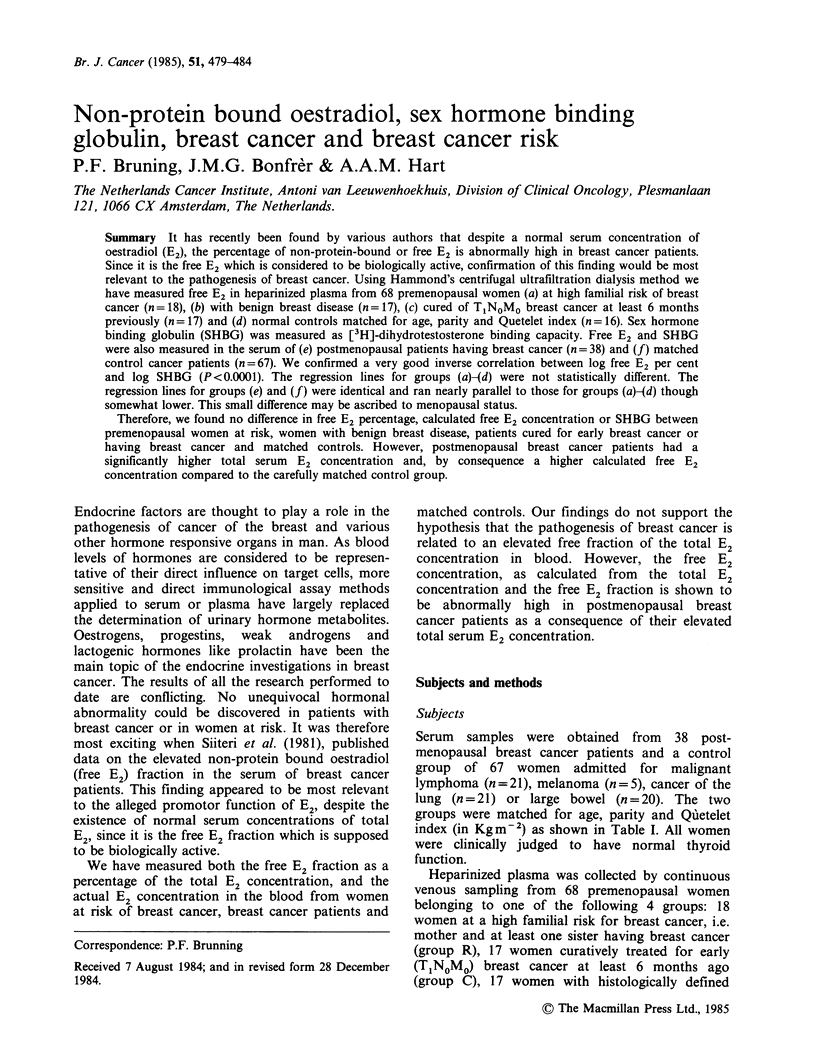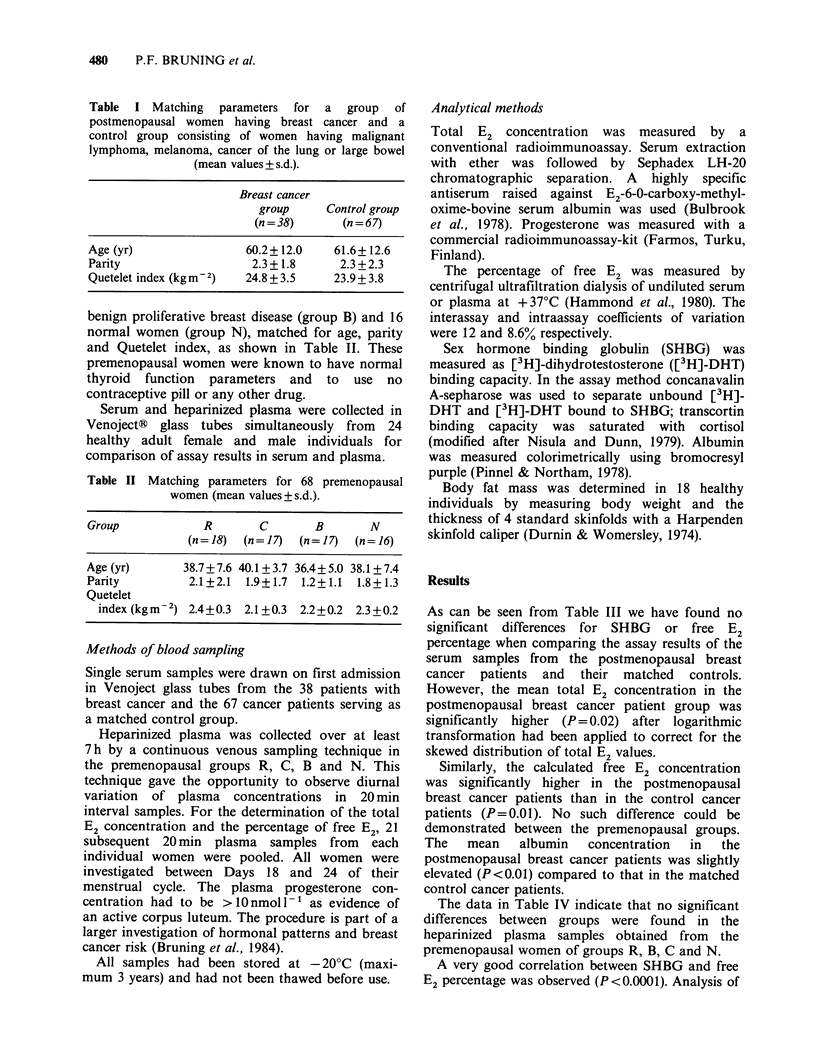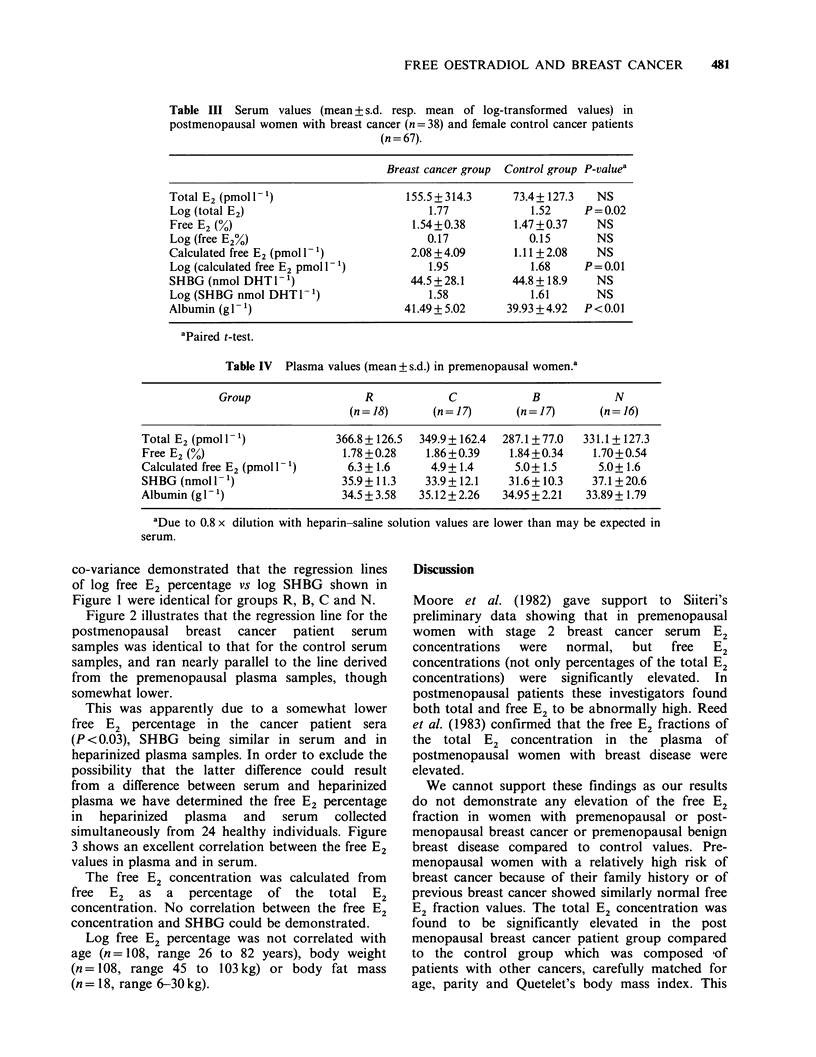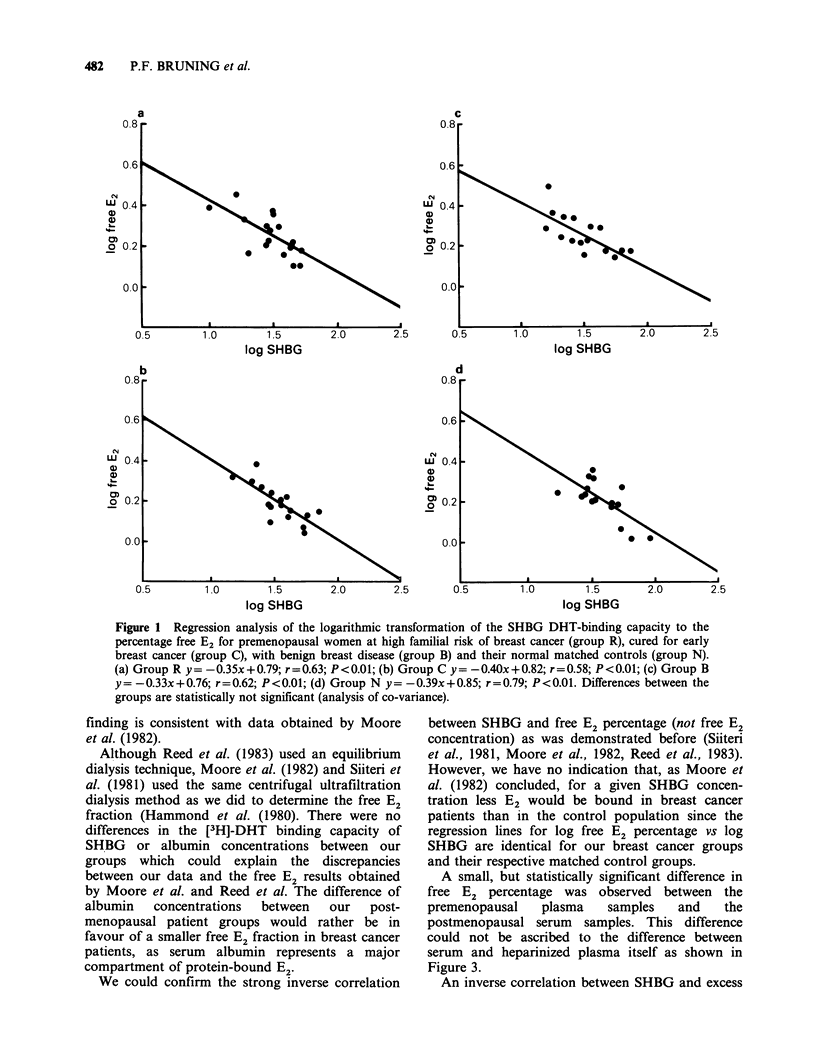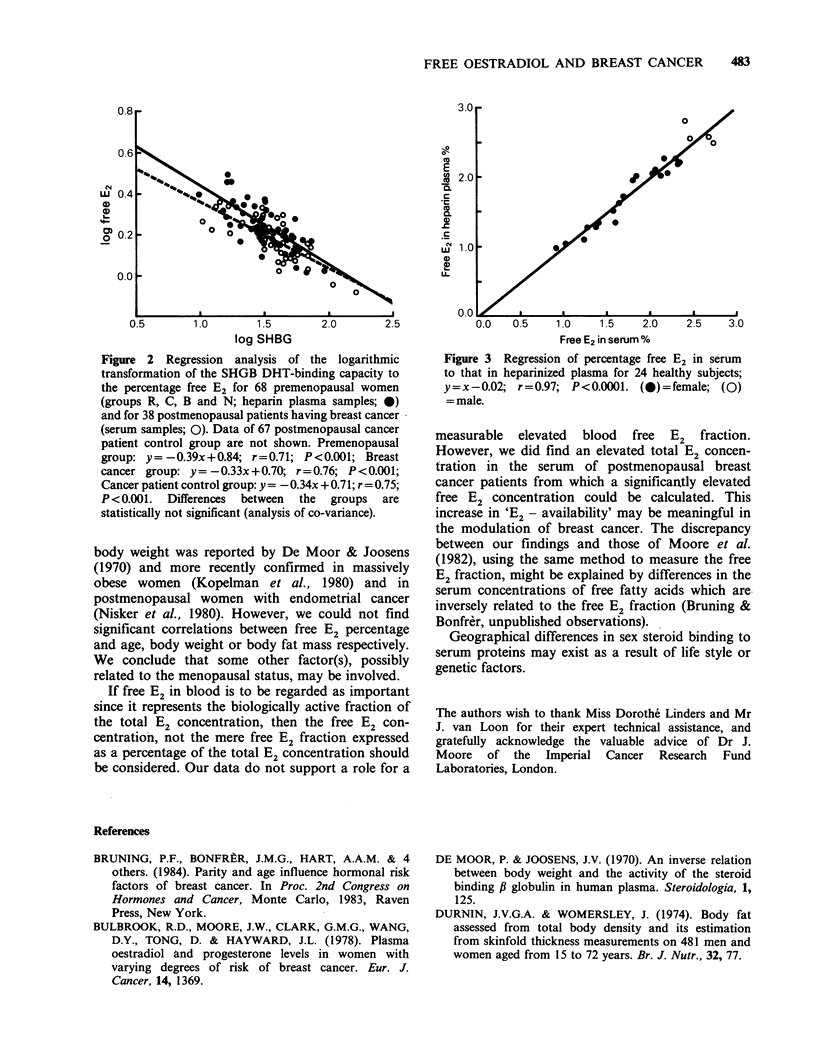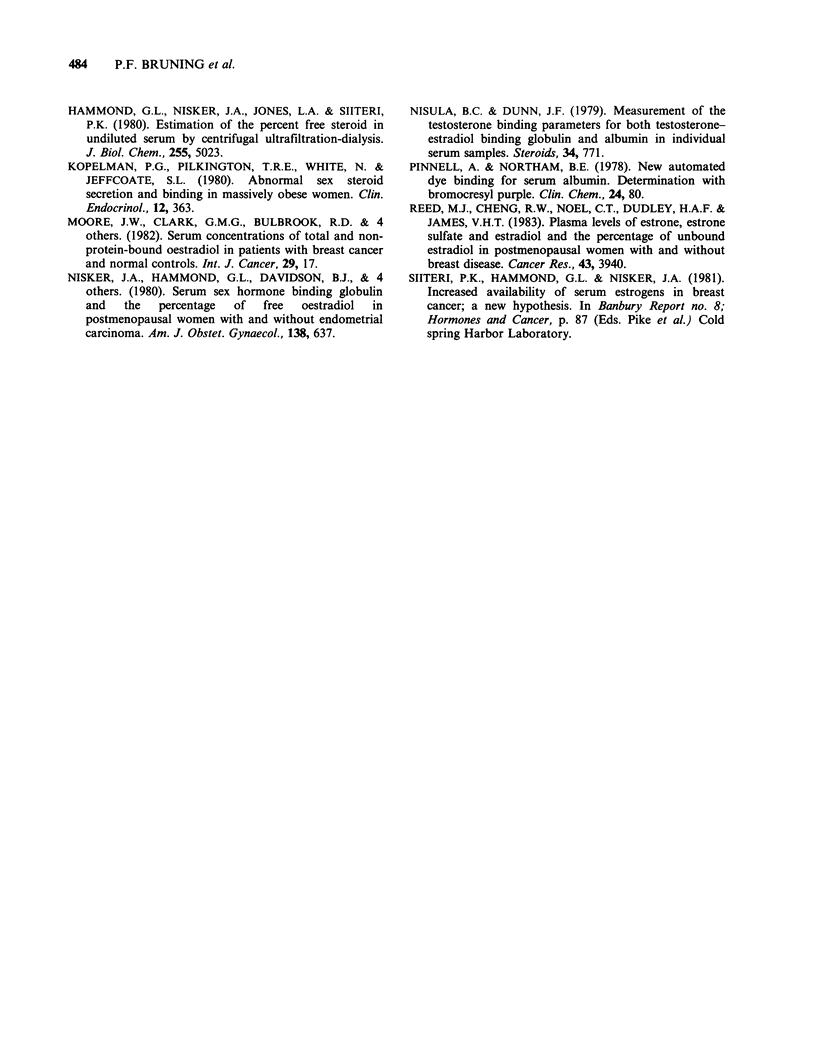# Non-protein bound oestradiol, sex hormone binding globulin, breast cancer and breast cancer risk.

**DOI:** 10.1038/bjc.1985.69

**Published:** 1985-04

**Authors:** P. F. Bruning, J. M. Bonfrèr, A. A. Hart

## Abstract

It has recently been found by various authors that despite a normal serum concentration of oestradiol (E2), the percentage of non-protein-bound or free E2 is abnormally high in breast cancer patients. Since it is the free E2 which is considered to be biologically active, confirmation of this finding would be most relevant to the pathogenesis of breast cancer. Using Hammond's centrifugal ultrafiltration dialysis method we have measured free E2 in heparinized plasma from 68 premenopausal women (a) at high familial risk of breast cancer (n = 18), (b) with benign breast disease (n = 17), (c) cured of T1N0M0 breast cancer at least 6 months previously (n = 17) and (d) normal controls matched for age, parity and Quetelet index (n = 16). Sex hormone binding globulin (SHBG) was measured as [3H]-dihydrotestosterone binding capacity. Free E2 and SHBG were also measured in the serum of (e) postmenopausal patients having breast cancer (n = 38) and (f) matched control cancer patients (n = 67). We confirmed a very good inverse correlation between log free E2 per cent and log SHBG (P less than 0.0001). The regression lines for groups (a)-(d) were not statistically different. The regression lines for groups (e) and (f) were identical and ran nearly parallel to those for groups (a)-(d) though somewhat lower. This small difference may be ascribed to menopausal status. Therefore, we found no difference in free E2 percentage, calculated free E2 concentration or SHBG between premenopausal women at risk, women with benign breast disease, patients cured for early breast cancer or having breast cancer and matched controls. However, postmenopausal breast cancer patients had a significantly higher total serum E2 concentration and, by consequence a higher calculated free E2 concentration compared to the carefully matched control group.


*Br. J. Cancer* (1985), **51**, 479–484

### Non-protein bound oestradiol, sex hormone binding globulin, breast cancer and breast cancer risk

P.F. Bruning, J.M.G. Bonfrèr & A.A.M. Hart

*The Netherlands Cancer Institute, Antoni van Leeuwenhoekhuis, Division of Clinical Oncology, Plesmanlaan 121, 1066 CX Amsterdam, The Netherlands.*

**Summary** It has recently been found by various authors that despite a normal serum concentration of oestradiol ($E_2$), the percentage of non-protein-bound or free $E_2$ is abnormally high in breast cancer patients. Since it is the free $E_2$ which is considered to be biologically active, confirmation of this finding would be most relevant to the pathogenesis of breast cancer. Using Hammond's centrifugal ultrafiltration dialysis method we have measured free $E_2$ in heparinized plasma from 68 premenopausal women (*a*) at high familial risk of breast cancer ($n=18$), (*b*) with benign breast disease ($n=17$), (*c*) cured of $T_1N_0M_0$ breast cancer at least 6 months previously ($n=17$) and (*d*) normal controls matched for age, parity and Quetelet index ($n=16$). Sex hormone binding globulin (SHBG) was measured as [$^3$H]-dihydrotestosterone binding capacity. Free $E_2$ and SHBG were also measured in the serum of (*e*) postmenopausal patients having breast cancer ($n=38$) and (*f*) matched control cancer patients ($n=67$). We confirmed a very good inverse correlation between log free $E_2$ per cent and log SHBG ($P<0.0001$). The regression lines for groups (*a*)–(*d*) were not statistically different. The regression lines for groups (*e*) and (*f*) were identical and ran nearly parallel to those for groups (*a*)–(*d*) though somewhat lower. This small difference may be ascribed to menopausal status.

Therefore, we found no difference in free $E_2$ percentage, calculated free $E_2$ concentration or SHBG between premenopausal women at risk, women with benign breast disease, patients cured for early breast cancer or having breast cancer and matched controls. However, postmenopausal breast cancer patients had a significantly higher total serum $E_2$ concentration and, by consequence a higher calculated free $E_2$ concentration compared to the carefully matched control group.

Endocrine factors are thought to play a role in the pathogenesis of cancer of the breast and various other hormone responsive organs in man. As blood levels of hormones are considered to be representative of their direct influence on target cells, more sensitive and direct immunological assay methods applied to serum or plasma have largely replaced the determination of urinary hormone metabolites. Oestrogens, progestins, weak androgens and lactogenic hormones like prolactin have been the main topic of the endocrine investigations in breast cancer. The results of all the research performed to date are conflicting. No unequivocal hormonal abnormality could be discovered in patients with breast cancer or in women at risk. It was therefore most exciting when Siiteri *et al.* (1981), published data on the elevated non-protein bound oestradiol (free $E_2$) fraction in the serum of breast cancer patients. This finding appeared to be most relevant to the alleged promotor function of $E_2$, despite the existence of normal serum concentrations of total $E_2$, since it is the free $E_2$ fraction which is supposed to be biologically active.

We have measured both the free $E_2$ fraction as a percentage of the total $E_2$ concentration, and the actual $E_2$ concentration in the blood from women at risk of breast cancer, breast cancer patients and matched controls. Our findings do not support the hypothesis that the pathogenesis of breast cancer is related to an elevated free fraction of the total $E_2$ concentration in blood. However, the free $E_2$ concentration, as calculated from the total $E_2$ concentration and the free $E_2$ fraction is shown to be abnormally high in postmenopausal breast cancer patients as a consequence of their elevated total serum $E_2$ concentration.

Correspondence: P.F. Brunning

Received 7 August 1984; and in revised form 28 December 1984.

### Subjects and methods

### Subjects

Serum samples were obtained from 38 postmenopausal breast cancer patients and a control group of 67 women admitted for malignant lymphoma ($n=21$), melanoma ($n=5$), cancer of the lung ($n=21$) or large bowel ($n=20$). The two groups were matched for age, parity and Qùetelet index (in $Kg\,m^{-2}$) as shown in Table I. All women were clinically judged to have normal thyroid function.

Heparinized plasma was collected by continuous venous sampling from 68 premenopausal women belonging to one of the following 4 groups: 18 women at a high familial risk for breast cancer, i.e. mother and at least one sister having breast cancer (group R), 17 women curatively treated for early ($T_1N_0M_0$) breast cancer at least 6 months ago (group C), 17 women with histologically defined

© The Macmillan Press Ltd., 1985

**Table I** Matching parameters for a group of postmenopausal women having breast cancer and a control group consisting of women having malignant lymphoma, melanoma, cancer of the lung or large bowel (mean values ± s.d.).

| | *Breast cancer group* (*n*=38) | *Control group* (*n*=67) |
|---|---|---|
| Age (yr) | 60.2 ± 12.0 | 61.6 ± 12.6 |
| Parity | 2.3 ± 1.8 | 2.3 ± 2.3 |
| Quetelet index ($kg\,m^{-2}$) | 24.8 ± 3.5 | 23.9 ± 3.8 |

benign proliferative breast disease (group B) and 16 normal women (group N), matched for age, parity and Quetelet index, as shown in Table II. These premenopausal women were known to have normal thyroid function parameters and to use no contraceptive pill or any other drug.

Serum and heparinized plasma were collected in Venoject® glass tubes simultaneously from 24 healthy adult female and male individuals for comparison of assay results in serum and plasma.

**Table II** Matching parameters for 68 premenopausal women (mean values ± s.d.).

| *Group* | *R* (*n*=18) | *C* (*n*=17) | *B* (*n*=17) | *N* (*n*=16) |
|---|---|---|---|---|
| Age (yr) | 38.7 ± 7.6 | 40.1 ± 3.7 | 36.4 ± 5.0 | 38.1 ± 7.4 |
| Parity | 2.1 ± 2.1 | 1.9 ± 1.7 | 1.2 ± 1.1 | 1.8 ± 1.3 |
| Quetelet index ($kg\,m^{-2}$) | 2.4 ± 0.3 | 2.1 ± 0.3 | 2.2 ± 0.2 | 2.3 ± 0.2 |

*Methods of blood sampling*

Single serum samples were drawn on first admission in Venoject glass tubes from the 38 patients with breast cancer and the 67 cancer patients serving as a matched control group.

Heparinized plasma was collected over at least 7 h by a continuous venous sampling technique in the premenopausal groups R, C, B and N. This technique gave the opportunity to observe diurnal variation of plasma concentrations in 20 min interval samples. For the determination of the total $E_2$ concentration and the percentage of free $E_2$, 21 subsequent 20 min plasma samples from each individual women were pooled. All women were investigated between Days 18 and 24 of their menstrual cycle. The plasma progesterone concentration had to be $>10\,nmol\,l^{-1}$ as evidence of an active corpus luteum. The procedure is part of a larger investigation of hormonal patterns and breast cancer risk (Bruning *et al.*, 1984).

All samples had been stored at −20°C (maximum 3 years) and had not been thawed before use.

*Analytical methods*

Total $E_2$ concentration was measured by a conventional radioimmunoassay. Serum extraction with ether was followed by Sephadex LH-20 chromatographic separation. A highly specific antiserum raised against $E_2$-6-0-carboxy-methyl-oxime-bovine serum albumin was used (Bulbrook *et al.*, 1978). Progesterone was measured with a commercial radioimmunoassay-kit (Farmos, Turku, Finland).

The percentage of free $E_2$ was measured by centrifugal ultrafiltration dialysis of undiluted serum or plasma at +37°C (Hammond *et al.*, 1980). The interassay and intraassay coefficients of variation were 12 and 8.6% respectively.

Sex hormone binding globulin (SHBG) was measured as [$^3$H]-dihydrotestosterone ([$^3$H]-DHT) binding capacity. In the assay method concanavalin A-sepharose was used to separate unbound [$^3$H]-DHT and [$^3$H]-DHT bound to SHBG; transcortin binding capacity was saturated with cortisol (modified after Nisula and Dunn, 1979). Albumin was measured colorimetrically using bromocresyl purple (Pinnel & Northam, 1978).

Body fat mass was determined in 18 healthy individuals by measuring body weight and the thickness of 4 standard skinfolds with a Harpenden skinfold caliper (Durnin & Womersley, 1974).

### Results

As can be seen from Table III we have found no significant differences for SHBG or free $E_2$ percentage when comparing the assay results of the serum samples from the postmenopausal breast cancer patients and their matched controls. However, the mean total $E_2$ concentration in the postmenopausal breast cancer patient group was significantly higher ($P = 0.02$) after logarithmic transformation had been applied to correct for the skewed distribution of total $E_2$ values.

Similarly, the calculated free $E_2$ concentration was significantly higher in the postmenopausal breast cancer patients than in the control cancer patients ($P = 0.01$). No such difference could be demonstrated between the premenopausal groups. The mean albumin concentration in the postmenopausal breast cancer patients was slightly elevated ($P < 0.01$) compared to that in the matched control cancer patients.

The data in Table IV indicate that no significant differences between groups were found in the heparinized plasma samples obtained from the premenopausal women of groups R, B, C and N.

A very good correlation between SHBG and free $E_2$ percentage was observed ($P < 0.0001$). Analysis of

**Table III** Serum values (mean ± s.d. resp. mean of log-transformed values) in postmenopausal women with breast cancer ($n=38$) and female control cancer patients ($n=67$).

| | *Breast cancer group* | *Control group* | *P-value*[a] |
|---|---|---|---|
| Total $E_2$ ($pmol\,l^{-1}$) | 155.5 ± 314.3 | 73.4 ± 127.3 | NS |
| Log (total $E_2$) | 1.77 | 1.52 | $P=0.02$ |
| Free $E_2$ (%) | 1.54 ± 0.38 | 1.47 ± 0.37 | NS |
| Log (free $E_2$%) | 0.17 | 0.15 | NS |
| Calculated free $E_2$ ($pmol\,l^{-1}$) | 2.08 ± 4.09 | 1.11 ± 2.08 | NS |
| Log (calculated free $E_2$ $pmol\,l^{-1}$) | 1.95 | 1.68 | $P=0.01$ |
| SHBG (nmol DHT $l^{-1}$) | 44.5 ± 28.1 | 44.8 ± 18.9 | NS |
| Log (SHBG nmol DHT $l^{-1}$) | 1.58 | 1.61 | NS |
| Albumin ($g\,l^{-1}$) | 41.49 ± 5.02 | 39.93 ± 4.92 | $P<0.01$ |

[a]Paired *t*-test.

**Table IV** Plasma values (mean ± s.d.) in premenopausal women.[a]

| *Group* | *R* ($n=18$) | *C* ($n=17$) | *B* ($n=17$) | *N* ($n=16$) |
|---|---|---|---|---|
| Total $E_2$ ($pmol\,l^{-1}$) | 366.8 ± 126.5 | 349.9 ± 162.4 | 287.1 ± 77.0 | 331.1 ± 127.3 |
| Free $E_2$ (%) | 1.78 ± 0.28 | 1.86 ± 0.39 | 1.84 ± 0.34 | 1.70 ± 0.54 |
| Calculated free $E_2$ ($pmol\,l^{-1}$) | 6.3 ± 1.6 | 4.9 ± 1.4 | 5.0 ± 1.5 | 5.0 ± 1.6 |
| SHBG ($nmol\,l^{-1}$) | 35.9 ± 11.3 | 33.9 ± 12.1 | 31.6 ± 10.3 | 37.1 ± 20.6 |
| Albumin ($g\,l^{-1}$) | 34.5 ± 3.58 | 35.12 ± 2.26 | 34.95 ± 2.21 | 33.89 ± 1.79 |

[a]Due to 0.8 × dilution with heparin–saline solution values are lower than may be expected in serum.

co-variance demonstrated that the regression lines of log free $E_2$ percentage *vs* log SHBG shown in Figure 1 were identical for groups R, B, C and N.

Figure 2 illustrates that the regression line for the postmenopausal breast cancer patient serum samples was identical to that for the control serum samples, and ran nearly parallel to the line derived from the premenopausal plasma samples, though somewhat lower.

This was apparently due to a somewhat lower free $E_2$ percentage in the cancer patient sera ($P<0.03$), SHBG being similar in serum and in heparinized plasma samples. In order to exclude the possibility that the latter difference could result from a difference between serum and heparinized plasma we have determined the free $E_2$ percentage in heparinized plasma and serum collected simultaneously from 24 healthy individuals. Figure 3 shows an excellent correlation between the free $E_2$ values in plasma and in serum.

The free $E_2$ concentration was calculated from free $E_2$ as a percentage of the total $E_2$ concentration. No correlation between the free $E_2$ concentration and SHBG could be demonstrated.

Log free $E_2$ percentage was not correlated with age ($n=108$, range 26 to 82 years), body weight ($n=108$, range 45 to 103 kg) or body fat mass ($n=18$, range 6–30 kg).

### Discussion

Moore *et al.* (1982) gave support to Siiteri's preliminary data showing that in premenopausal women with stage 2 breast cancer serum $E_2$ concentrations were normal, but free $E_2$ concentrations (not only percentages of the total $E_2$ concentrations) were significantly elevated. In postmenopausal patients these investigators found both total and free $E_2$ to be abnormally high. Reed *et al.* (1983) confirmed that the free $E_2$ fractions of the total $E_2$ concentration in the plasma of postmenopausal women with breast disease were elevated.

We cannot support these findings as our results do not demonstrate any elevation of the free $E_2$ fraction in women with premenopausal or postmenopausal breast cancer or premenopausal benign breast disease compared to control values. Premenopausal women with a relatively high risk of breast cancer because of their family history or of previous breast cancer showed similarly normal free $E_2$ fraction values. The total $E_2$ concentration was found to be significantly elevated in the post menopausal breast cancer patient group compared to the control group which was composed of patients with other cancers, carefully matched for age, parity and Quetelet's body mass index. This

**Figure 1** Regression analysis of the logarithmic transformation of the SHBG DHT-binding capacity to the percentage free $E_2$ for premenopausal women at high familial risk of breast cancer (group R), cured for early breast cancer (group C), with benign breast disease (group B) and their normal matched controls (group N). (a) Group R $y=-0.35x+0.79$; $r=0.63$; $P<0.01$; (b) Group C $y=-0.40x+0.82$; $r=0.58$; $P<0.01$; (c) Group B $y=-0.33x+0.76$; $r=0.62$; $P<0.01$; (d) Group N $y=-0.39x+0.85$; $r=0.79$; $P<0.01$. Differences between the groups are statistically not significant (analysis of co-variance).

finding is consistent with data obtained by Moore *et al.* (1982).

Although Reed *et al.* (1983) used an equilibrium dialysis technique, Moore *et al.* (1982) and Siiteri *et al.* (1981) used the same centrifugal ultrafiltration dialysis method as we did to determine the free $E_2$ fraction (Hammond *et al.* (1980). There were no differences in the [$^3$H]-DHT binding capacity of SHBG or albumin concentrations between our groups which could explain the discrepancies between our data and the free $E_2$ results obtained by Moore *et al.* and Reed *et al.* The difference of albumin concentrations between our post-menopausal patient groups would rather be in favour of a smaller free $E_2$ fraction in breast cancer patients, as serum albumin represents a major compartment of protein-bound $E_2$.

We could confirm the strong inverse correlation between SHBG and free $E_2$ percentage (*not* free $E_2$ concentration) as was demonstrated before (Siiteri *et al.*, 1981, Moore *et al.*, 1982, Reed *et al.*, 1983). However, we have no indication that, as Moore *et al.* (1982) concluded, for a given SHBG concentration less $E_2$ would be bound in breast cancer patients than in the control population since the regression lines for log free $E_2$ percentage *vs* log SHBG are identical for our breast cancer groups and their respective matched control groups.

A small, but statistically significant difference in free $E_2$ percentage was observed between the premenopausal plasma samples and the postmenopausal serum samples. This difference could not be ascribed to the difference between serum and heparinized plasma itself as shown in Figure 3.

An inverse correlation between SHBG and excess

**Figure 2** Regression analysis of the logarithmic transformation of the SHGB DHT-binding capacity to the percentage free $E_2$ for 68 premenopausal women (groups R, C, B and N; heparin plasma samples; ●) and for 38 postmenopausal patients having breast cancer (serum samples; ○). Data of 67 postmenopausal cancer patient control group are not shown. Premenopausal group: $y=-0.39x+0.84$; $r=0.71$; $P<0.001$; Breast cancer group: $y=-0.33x+0.70$; $r=0.76$; $P<0.001$; Cancer patient control group: $y=-0.34x+0.71$; $r=0.75$; $P<0.001$. Differences between the groups are statistically not significant (analysis of co-variance).

**Figure 3** Regression of percentage free $E_2$ in serum to that in heparinized plasma for 24 healthy subjects; $y=x-0.02$; $r=0.97$; $P<0.0001$. (●) = female; (○) = male.

body weight was reported by De Moor & Joosens (1970) and more recently confirmed in massively obese women (Kopelman *et al.*, 1980) and in postmenopausal women with endometrial cancer (Nisker *et al.*, 1980). However, we could not find significant correlations between free $E_2$ percentage and age, body weight or body fat mass respectively. We conclude that some other factor(s), possibly related to the menopausal status, may be involved.

If free $E_2$ in blood is to be regarded as important since it represents the biologically active fraction of the total $E_2$ concentration, then the free $E_2$ concentration, not the mere free $E_2$ fraction expressed as a percentage of the total $E_2$ concentration should be considered. Our data do not support a role for a measurable elevated blood free $E_2$ fraction. However, we did find an elevated total $E_2$ concentration in the serum of postmenopausal breast cancer patients from which a significantly elevated free $E_2$ concentration could be calculated. This increase in '$E_2$ – availability' may be meaningful in the modulation of breast cancer. The discrepancy between our findings and those of Moore *et al.* (1982), using the same method to measure the free $E_2$ fraction, might be explained by differences in the serum concentrations of free fatty acids which are inversely related to the free $E_2$ fraction (Bruning & Bonfrèr, unpublished observations).

Geographical differences in sex steroid binding to serum proteins may exist as a result of life style or genetic factors.

The authors wish to thank Miss Dorothé Linders and Mr J. van Loon for their expert technical assistance, and gratefully acknowledge the valuable advice of Dr J. Moore of the Imperial Cancer Research Fund Laboratories, London.

### References

BRUNING, P.F., BONFRÈR, J.M.G., HART, A.A.M. & 4 others. (1984). Parity and age influence hormonal risk factors of breast cancer. In *Proc. 2nd Congress on Hormones and Cancer,* Monte Carlo, 1983, Raven Press, New York.

BULBROOK, R.D., MOORE, J.W., CLARK, G.M.G., WANG, D.Y., TONG, D. & HAYWARD, J.L. (1978). Plasma oestradiol and progesterone levels in women with varying degrees of risk of breast cancer. *Eur. J. Cancer,* **14,** 1369.

DE MOOR, P. & JOOSENS, J.V. (1970). An inverse relation between body weight and the activity of the steroid binding $\beta$ globulin in human plasma. *Steroidologia,* **1,** 125.

DURNIN, J.V.G.A. & WOMERSLEY, J. (1974). Body fat assessed from total body density and its estimation from skinfold thickness measurements on 481 men and women aged from 15 to 72 years. *Br. J. Nutr.,* **32,** 77.

HAMMOND, G.L., NISKER, J.A., JONES, L.A. & SIITERI, P.K. (1980). Estimation of the percent free steroid in undiluted serum by centrifugal ultrafiltration-dialysis. *J. Biol. Chem.*, **255**, 5023.

KOPELMAN, P.G., PILKINGTON, T.R.E., WHITE, N. & JEFFCOATE, S.L. (1980). Abnormal sex steroid secretion and binding in massively obese women. *Clin. Endocrinol.*, **12**, 363.

MOORE, J.W., CLARK, G.M.G., BULBROOK, R.D. & 4 others. (1982). Serum concentrations of total and non-protein-bound oestradiol in patients with breast cancer and normal controls. *Int. J. Cancer*, **29**, 17.

NISKER, J.A., HAMMOND, G.L., DAVIDSON, B.J., & 4 others. (1980). Serum sex hormone binding globulin and the percentage of free oestradiol in postmenopausal women with and without endometrial carcinoma. *Am. J. Obstet. Gynaecol.*, **138**, 637.

NISULA, B.C. & DUNN, J.F. (1979). Measurement of the testosterone binding parameters for both testosterone–estradiol binding globulin and albumin in individual serum samples. *Steroids*, **34**, 771.

PINNELL, A. & NORTHAM, B.E. (1978). New automated dye binding for serum albumin. Determination with bromocresyl purple. *Clin. Chem.*, **24**, 80.

REED, M.J., CHENG, R.W., NOEL, C.T., DUDLEY, H.A.F. & JAMES, V.H.T. (1983). Plasma levels of estrone, estrone sulfate and estradiol and the percentage of unbound estradiol in postmenopausal women with and without breast disease. *Cancer Res.*, **43**, 3940.

SIITERI, P.K., HAMMOND, G.L. & NISKER, J.A. (1981). Increased availability of serum estrogens in breast cancer; a new hypothesis. In *Banbury Report no. 8; Hormones and Cancer*, p. 87 (Eds. Pike *et al.)* Cold spring Harbor Laboratory.